# Layered-Fabric Materiality Fibre Reinforced Polymers (L-FMFRP): Hysteretic Behavior in Architectured FRP Material

**DOI:** 10.3390/polym14061141

**Published:** 2022-03-12

**Authors:** Arielle Blonder, Maurizio Brocato

**Affiliations:** Laboratoire GSA, Ecole National Supérieure d’Architecture Paris-Malaquais, Université PSL, 75006 Paris, France; maurizio.brocato@paris-malaquais.archi.fr

**Keywords:** fiber composites, FRP, architecture, architectured materials, hysteresis

## Abstract

L_FMFRP is an architectural fiber composite surface element with an airy internal structure and variable section. This architectured material is the product of an alternative design and fabrication process that integrates *fabric materiality*, suggesting moldless shaping of the material through pleating and layering. Initial study of the mechanical properties of the element showed a structural behavior that would satisfy the requirement for schematic architectural cladding configurations, indicating a unique hysteretic behavior of the material. This paper further investigates the hysteretic capacities of L-FMFRP, examining the behavior under repeated loading and the effect of its internal material architecture. Parallels to entangled materials are suggested for a deeper understanding of the phenomenon, and the potential future application as an energy-absorbent material for façade cladding is outlined.

## 1. Introduction

Fiber-reinforced polymers (FRPs) are the composite outcome of advanced fibers and polymer resins, making a family of high-performance materials that offers high strength-to-weight ratio, durability and versatility. In the past decades, numerous types of FRPs have been developed and introduced into extensive use across industries, from aeronautics and space, to infrastructure, automotive and consumer products. In the construction industry, FRPs are widely used for civil infrastructure [1], rehabilitation and reinforcement, light and moveable constructions, footbridges, profiles and decks, under various forms and fabrication processes [2]. Their architectural use is re-emerging [3], expanding from experimental pavilions and academic research [4] to wider commercial applications by world-leading architects [5].

Elements of FRPs are traditionally manufactured based on rigid molds. A compact laminate is made by pressing a number of fiber layers, mainly under the form of textile fabrics, over the mold. The composite piece is shaped by the mold’s morphology, assuring conformity to the requested designed shape as well as material homogeneity [6]. While being substantial to applications in fields such as the automotive, security or aeronautics sectors, the dependence on rigid molds can be restrictive in the case of architectural applications [7]. The typical size-of-element and its one-off nature, together with contemporary architectural practices that promote complex morphologies and high variability, stand in contradiction to the practice and economy of mold-based manufacturing. Standard industrial composite-forming processes therefore represent a significant factor in the barrier to the wider application of the material in the architectural field [8]. This barrier is reflected in numerous contemporary research projects, seeking for alternative, adaptive, reconfigurable moldless forming processes and material systems in architecture [9,10,11]. In fiber composites, a major approach to alternative shaping is tackled at the fiber-bundle level, through robotic filament winding fabrication [12,13].

**Figure 1:**
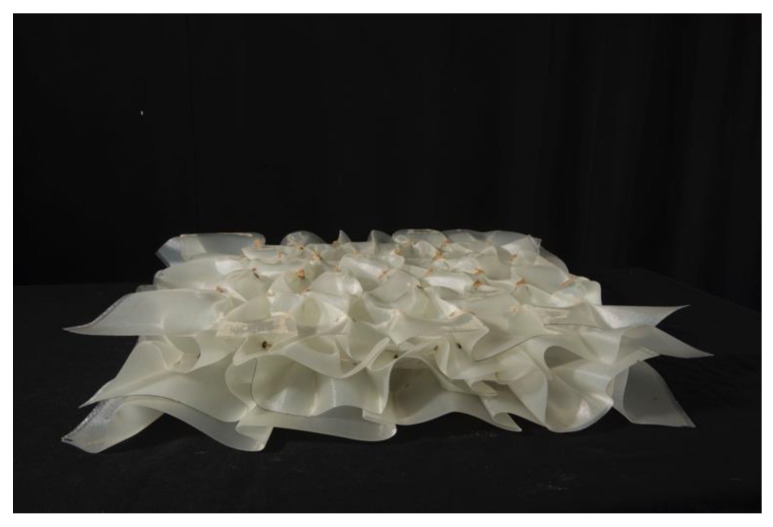
Layered FM-FRP material system (L-FMFRP) resulting in architectured material shaped as a thick panel.

This research tackles the alternative shaping of FRPs on the fabric level, enhancing the textile qualities of the fiber constituent of the composite. The term “*fabric materiality* FRP” (FM-FRP) was coined to represent the integration of textile-related practices, techniques, design paradigms and material qualities into the world of fiber composites, as novel FRP material systems [14]. FM-FRP typically would inherit key assets from the world of textiles, such as parametric variability, self-organization and resilience, resulting in novel architectural material systems. Fabric manipulations and self-organization capacities substitute the extensive use of molds, thus suggesting alternative architectural outcomes. The FM-FRP material system that was developed based on the layering of pleated surfaces resulted in an airy element with a variable and intricate section; blurring the boundaries between structure and matter, it resembles a thick panel [14] (Figure 1). The combination of matter and voids as an internal material structure and the creation of porous systems with enhanced capacities for multifunctional applications [15] is currently of high interest, developing *architectured materials*, or *metamaterials* [16]. The voids can be distributed periodically (such as in lattices [17]) or in relative disorder (such as in entangled materials [18]).

In the field of composites, the integration of voids in the material architecture is mainly applied as tubular hollow composites, based on techniques such as braiding, knitting, or spacer [19]. Such hollow-structured elements can also be incorporated as nested inserts in more complex sandwich structures when seeking to further optimize performance and light weight [20]. Out of the various applications of tubular composites, from sports equipment and printing rollers to rocket structures and helicopter landing gears, various applications make use of the crashworthiness and energy absorption properties of hollow composites, as energy-absorbent composite structures (EACS). Composites can absorb a substantial amount of energy per unit mass in comparison to metals. Other advantages of EACS include higher strength, lower weight, higher specific-stiffness, better potential in terms of vibration control and noise reduction [21]. Due to the brittle nature of composites, the energy is absorbed in FRP structures mostly through the conversion of kinetic energy to a form of deformation absorbed energy—a complex fracture mechanism of cracking, delamination and fiber breakage [21]. The level of energy absorption is affected by a variety of parameters of geometrical and material nature, depending on the fiber architecture as well as the resin and matrix material characteristics [22]. The unique energy-absorbing qualities of composite structures gradually make them a preferred choice for crashworthiness applications, along with increasing research interest and technological progress in the field [21]. 

The preliminary testing of L-FMFRP material demonstrated its general suitability for service as an architectural façade element [23]. In particular, compression tests revealed a capacity of the panel for large quasi-elastic deformation and a good recovery of the material after extensive compression, indicating the potential energy-absorption qualities of the material. 

In the past two decades, there has been a growing concern for safety and security in the built environment. From the scale of urban planning to material design, efforts are being invested in the creation of resilient built environments that would actively contribute toward facing the challenges of terrorism and of natural disasters [24]. The growing threat of terrorist attacks in city centers urges the development of blast-resistant façade systems [25] and energy-absorbent material systems [26]. In parallel, climate change accentuates the occurrence of natural disasters such as cyclones, hurricanes and typhoons, causing tremendous casualties and physical damage. Studies have shown that the windborne debris of storms, generated either by unsecured items or by the progressive failure of the built environment, plays a major part in causing damage. Failure of the façade elements prove to be hazardous for the surroundings, the structure itself and its occupants, and can ultimately lead to the general failure of the structure by changing the internal–external balance of air pressure [27]. Aiming to improve performance with regard to both natural disasters and terrorist attacks, façade materials are re-evaluated with regard to updated codes and regulations [28], and structural concepts such as sacrificial façade systems are developed [29]. Fiber composites can potentially answer the need for cladding systems that are lightweight and perform as efficient energy absorbers [26]. The typical plastic deformation of extensive micro-cracking, rather than general buckling failure, together with the high ratio of density to flexural stiffness make FRP materials a potentially suitable domain for the development of such solutions [30].

Following the initial indications for the potential energy-absorption capacities and interesting properties of L-FMFRP under compression, three issues were defined for further investigation through compressive tests:

(a) Test the unloading behavior: verify whether or not the unloading curve is inversely similar to the loading curve and identify the elasto-plastic characteristics of the material behavior.
(b) Test repeated cycles: observe the element during multiple loading–unloading cycles to identify a possible drift in the cyclic curves.
(c) Test various folding patterns: investigate the effect of different folding patterns on the overall elasto-plastic behavior of the element.

Two experimental campaigns were carried out for the investigation of the above three issues. The main results of these tests are the observation of a phenomenon of hysteresis and some correlations between the shape of the loading–unloading paths and the meso-architecture of the specimens. 

## 2. Materials and Methods

### 2.1. Materials and Sample Preparation

Testing was realized on L-FMFRP specimens, all made of fiberglass–epoxy prepreg 0/90 satin weave of 300 g/m^2^: Prepreg E-glass 7781: fabric thickness 0.23 mm, weave pattern 8HS; resin content 30% +/−3%, Tg 124°. 

**Figure 2:**
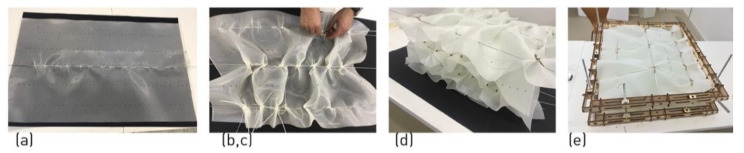
L-FMFRP fabrication process: (**a**) introducing metal rods in pre-cut holes in the fabric; (**b**) contracting the fabric at points along the rod; (**c**) assuring the local contractions with a temporary string; (**d**) super-imposing the manipulated fabric; (**e**) contracting the layered assembly with a jig for oven curing.

The samples were fabricated according to a protocol that was developed in the framework of previous research [23], which consists of the pleating of single sheets and their stacking with light peripheral constraint, in order to form the equivalent of a laminate, a volumetric element (Figure 2): 

Pleating of the single sheet:

(a) Perforation of the prepreg sheet according to pattern (number and placing of lines over the sheet, spacing of holes along the lines).
(b) Sliding metal rods into the perforated lines of holes to serve as pleating guides.
(c) Gathering the pleats by temporary knots along the pleating guides (the metal rod).

Forming the ‘laminate’:

(a) Stacking the pleated sheet by superposition.
(b) Constraining the assembly by a jig.
(c) Curing.

All samples were composed of two prepreg layers, 600/200 mm each. The stacked assembly was oven-cured at a temperature of 125 °C for 2 h.

#### 2.1.1. First Experimental Campaign

**Figure 3:**
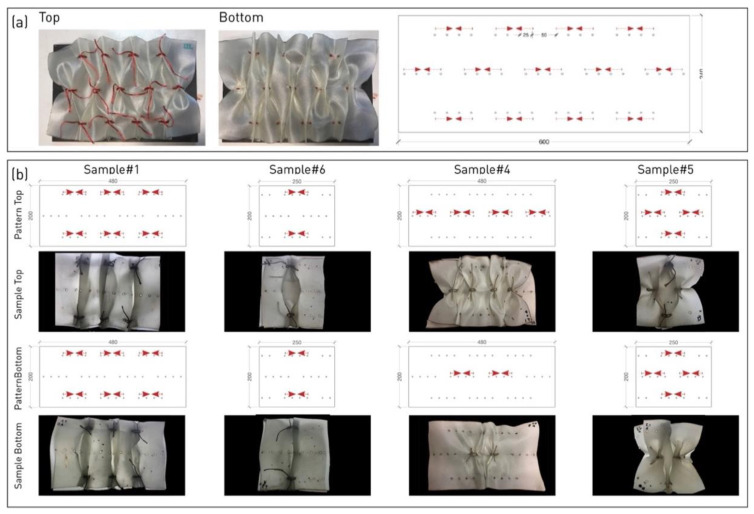
Samples of two experimental campaigns. (**a**) First campaign: typical sample top (left), and bottom view (middle), pleating pattern for both layers (right). (**b**) Second campaign: four tested samples, views and pleating patterns of top and bottom layers.

The testing was carried over three samples of identical pattern and similar internal structure (Figure 3a). The pleating pattern is composed of three rows, with metal rods aligning five pleats (gathering points) along its central row. As the product is not molded, and the manipulated layers are just lightly compressed in the curing jig, the different samples exhibit variations; the final dimensions of the panel were averagely 300–320 L/190 W/40–55 H (mm).

#### 2.1.2. Second Experimental Campaign

Testing was carried over four samples, each with a different pleating pattern. Two pleat types were used: a ‘simple pleat’ pinched along two rods (top and bottom) and a ‘full pleat’ pinched along three rods (top, center, bottom) (Figure 3b). Each sample comprised two layers of similar pleating type and a variable number of pleats. The final dimensions of the samples varied according to the pleat types (Table 1). 

### 2.2. Mechanical Testing

#### 2.2.1. First Experimental Campaign

The testing was realized in an electromechanical tension–compression MTS testing machine of 100 kN capacity, with the samples placed between two steel plates (20 mm thickness) for levelling and load distribution over the surface. An average of 20 loading cycles were realized per sample, at constant displacement rate (between 8 and 40 mm/min, varying between test sets). The maximal load was averagely 3000 N (excluding the first cycles of the setting and the last cycles of the rupture).

#### 2.2.2. Second Experimental Campaign

**Figure 4:**
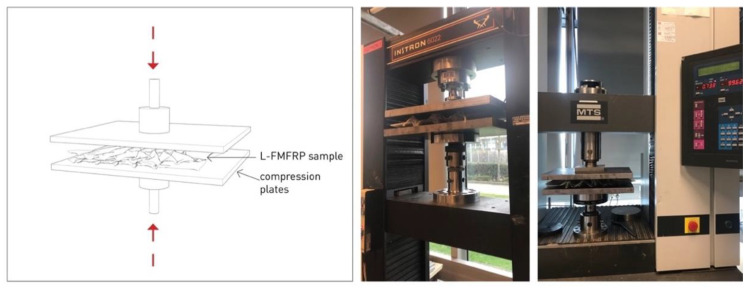
Experiment setup: sample positioned between two steel levelling plates.

The testing was realized in an electromechanical tension–compression Instron testing machine of 10 kN capacity, with the samples placed between two steel plates (20 mm thickness) for levelling and load distribution over the surface (Figure 4).

## 3. Results

### 3.1. Preliminary Testing

**Figure 5:**
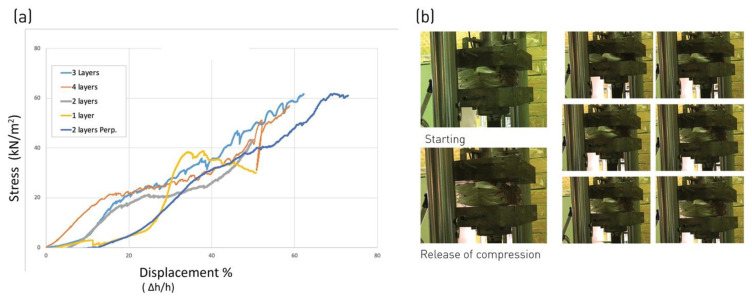
Compressive tests, distributed load. (**a**) Stress-displacement curves over five samples of different layer composition. (**b**) Sample during test: application of force and release of compression stress.

A preliminary study of L-FMFRP’s structural characteristics was carried out in the framework of previous research, testing the surface element under tension, compression and bending. The results showed a structural behavior that would satisfy the requirements for different theoretical schematic architectural cladding applications [23]. The compression test set (distributed load perpendicular to the panel surface) indicated a capacity of the panel for large quasi-elastic deformations. The generation of large displacement under loading (compressing the system to less than half of its initial thickness) did not lead to failure, but for the appearance of a few minor localized cracks. Furthermore, the initial thickness of the panel was almost entirely recovered after unloading (Figure 5). The observed behavior indicated the potential properties of hysteresis, and the capacity of energy absorption of the system, which led to the following two experimental campaigns.

### 3.2. First Experimental Campaign: Cyclic Loading

**Figure 6:**
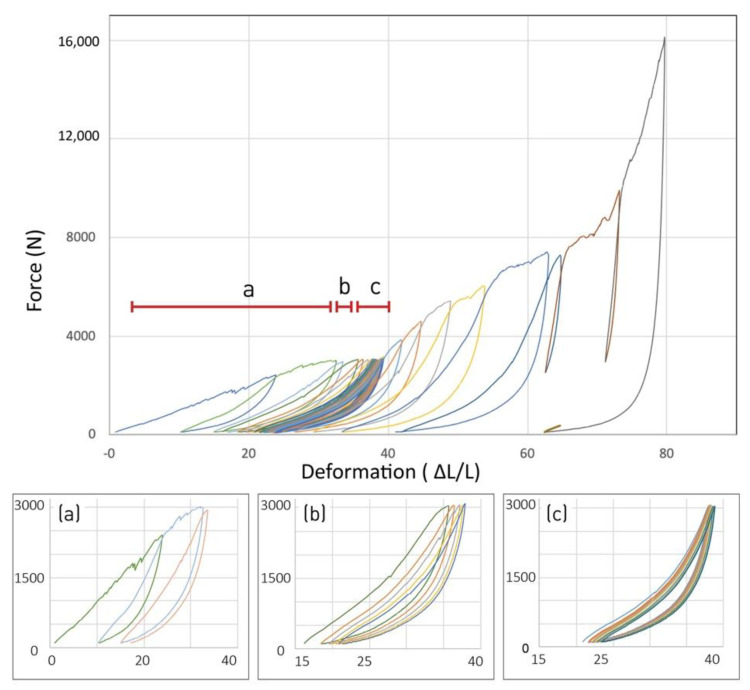
Typical loading/unloading curve: evolution along test sets. Sections in detail below: (**a**) settling, (**b**) first hysteretic cycles, (**c**) steady repeated cycles (Experimental campaign1_sample01).

The results of the first experimental campaign showed a significant difference between the loading and the unloading curves. When plotting the force against deformation, the two curves form a loop with an area enclosed between the curves (Figure 6). The loading curve shows positive hardening (a greater force required as the deformation increases) and the difference between the curves indicates a different path taken by the material upon loading/unloading. This typical curve, resembling exponential progression, is different from the curves that were plotted in the initial compression tests (operated on samples of various architectures (Figure 5)), which showed a more linear progression of force as the deformation advances. Looking at the first cycles of compression in the repeated loading of the current experiments, we see an evolution of the curve, from a quasi-linear slope in the first compression cycle, which resembles the previous initial compression tests, to the exponential type of curve by the third compression cycle. The evolution between the test sets of a single sample shows a settling of the material during the first loading cycles, and as the cycles are repeated, the response gradually stabilizes (Figure 6).

**Figure 7:**
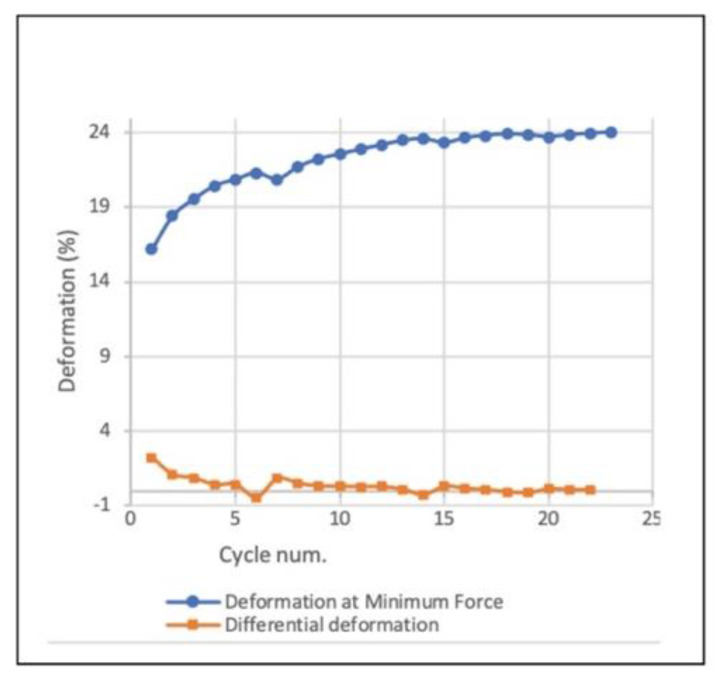
Accumulated irreversible deformation across loading cycles: plotted deformation at minimal applied force (blue) and differential deformation (orange).

Increasing the applied force beyond the previously achieved threshold generates an irreversible plastic deformation, visible in the dented curve section. The repeated loading thereafter within the newly achieved threshold keeps a reversible elastic behavior with smooth curves. A small shift in the zero-state at each loading cycle is noticeable (within a similar threshold), showing an accumulated minimal irreversible deformation (about 6% overall). However, as the number of cycles increases under the same threshold, the accumulated irreversible deformation decreases (Figure 7).

**Figure 8:**
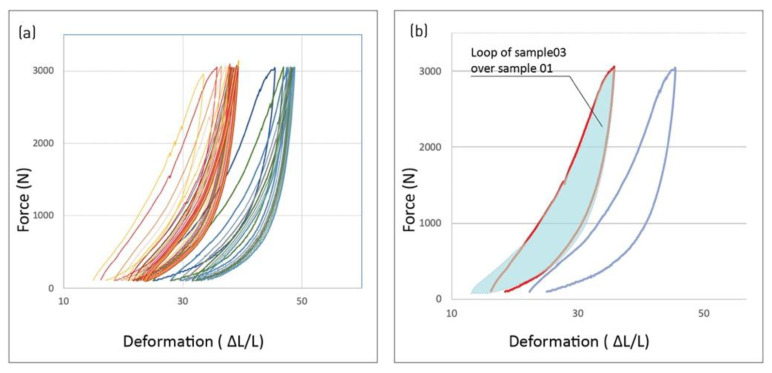
Similarity of loading–unloading loops between two samples of identical pleating pattern. (**a**) Repeated loading of sample 01 (red) and sample 03 (blue). (**b**) Comparison of loops between samples: enclosed area of sample 03 (blue stain) superimposed over loop curve of sample 01 (red).

A similar loop curve appears among all three samples, despite their relative differences due to manual fabrication and material self-organization. A superposition of loops of different samples under identical loading thresholds strongly resembles the curves and the enclosed area between the loading–unloading (Figure 8). 

### 3.3. Second Experimental Campaign: Pleating Pattern

**Figure 9:**
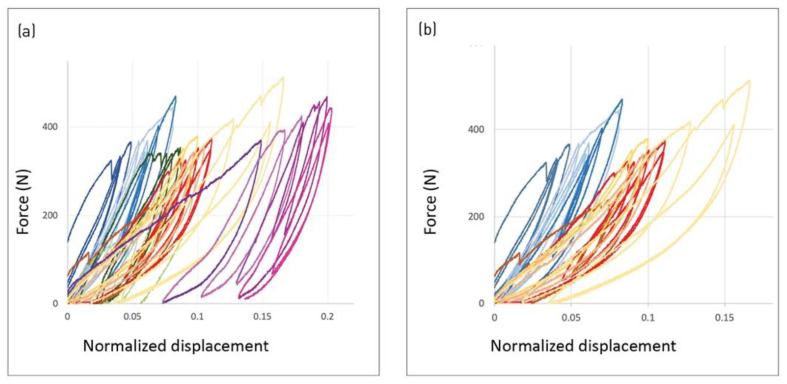
Effect of pleat types force plotted against normalized deformation and grouped by color shades. (**a**) All samples combined: one simple pleat (blue); one full pleat (red/yellow); three simple pleats (green); three full pleats (purple). (**b**) Comparison between simple and full pleat types: simple pleat (blue), showing steeper curves and smaller enclosed area in path; full pleat (red), showing larger enclosed area and shallower curves.

The results of the second experimental campaign show the variation of the hysteretic loop between the samples of different pleating patterns. General similarity remains between the samples, as all demonstrate the hysteretic loop. However, the enclosed area of the loops as well as the overall steepness of the curves vary between samples. The plotting force against the normalized displacement of all samples combined shows that the samples of the full pleats (red, purple) go through larger relative deformation compared to the simple pleats (blue, green). Accordingly, the enclosed area of their loops is larger, showing a stronger difference between the loading and unloading paths (Figure 9). A comparative look at two samples of a single pleat in the two configurations (simple/full) shows the steeper curves of the single pleat compared to the full one, as well as a larger area enclosed in the loading–unloading loop.

**Figure 10:**
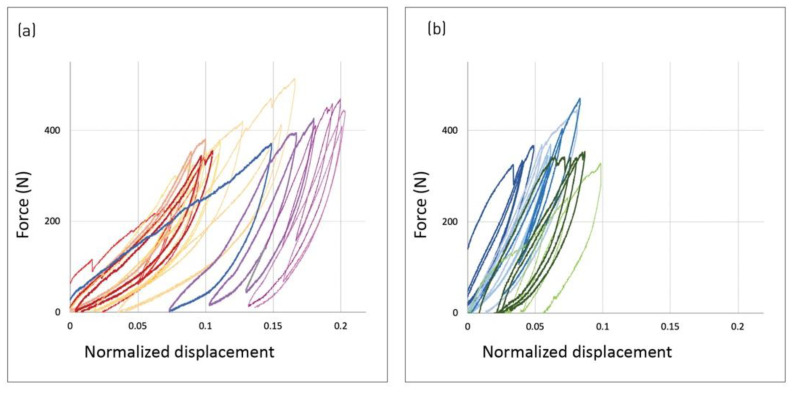
Effect of pleat number—increasing the number of pleats reduces stiffness and increases energy absorption. (**a**) Full pleats: single (purple) and triple (red). (**b**) Simple pleats: single (blue) and triple (green).

The comparison between single and triple pleats of the same kind shows a similar behavior for both simple and full pleats (Figure 10). The higher displacement values for a larger number of pleats indicate lower stiffness. The enclosed area of the hysteresis loops rises with the number of pleats, indicating higher energy absorption. 

## 4. Discussion

The experiments show a loop of a defined deformation cycle (loading and unloading curves) enclosing an area, which indicates a loss of elastic energy in the process, allegedly attributed to friction. Such behavior is known as hysteresis, occurring in a variety of materials, structures and systems, where the dependence of a physical system upon its history is expressed as a non-linear behavior with a retardation of the effect when external forces acting upon the system are changed. This phenomenon is typically associated with materials of visco-elastic behavior such as elastomers [31], and is not particularly typical of FRP, where no intrinsic dissipation mechanisms exist in statics before failure [32]. On the contrary, when in dynamics, FRP may have damping capacities by dissipating some elastic energy through the visco-elastic behavior of their resin, or by various fracture mechanisms [20]. 

The similarity of load–unload loops between the different samples clearly demonstrates that the hysteretic behavior is a typical property of the L-FMFRP architectured material. While the samples show significant variations in their specific material configuration, due to manual manufacturing and material self-organization, the strong similarity of hysteretic loops between the samples indicates that this property is not sensitive to specific internal configuration (Figure 8). Rather, it seems to be dictated by the overall material architecture, i.e., the pleating pattern and the connections between the layers. A parallel can be made to other architectured materials with randomly disordered structure at the meso scale, such as entangled materials [33] or the jamming of aggregate systems [18]. Such systems demonstrate hysteretic behaviors that depend on their internal architectural parameters. Parameters such as the number of connections between the discrete components of the system (such as fibers or granules), their orientation or aspect ratio determine the level of energy dissipation of the system [34]. 

The effect of different parameters of the material architectures of L-FMFRP and its behavior can be initially observed through the comparison between different pleating patterns (campaign 02). Although the isolation and modelling of the effect of each parameter requires further study, the difference between the full and simple pleats is noticeable in the typical loading–unloading paths (Figure 9). The two different patterns generate surfaces of different geometrical complexity; the manipulation of the simple pleat develops into a barrel-like curved surface, and the full pleat develops into conical-type formations with a higher degree of geometrical complexity. The elements generated by patterns that are more geometrically complex show reduced stiffness (indicated by the reduced overall steepness of the loading curve). The connection between the folding complexity of the surface and its reduced stiffness could be compared to the behavior of knitted hollow composites, where the elastic stage of the compression curve corresponds to the flattening and ovalization of the hollow channels, and is correlated with the channel’s radius of curvature [24]. Whether the model describing the compression curve of hollow composites could be suitable for our case should be further investigated as well (transitioning from elastic flattening of the structure to deformation at the joining points, and finally the densification of fibers and load transfer to matrix). In parallel to reduced stiffness, a larger area is enclosed in the hysteretic loop of the load–unload curves, with increased surface complexity. This is visible both in the comparison between simple and full pleats, as well as between surfaces of single or multiple pleats. This could be explained by the role friction plays in the hysteretic process, determined by parameters such as the number of contact points, the tangency of surfaces and areas of contact, which are generated by the pleating pattern and will be further investigated. A parallel can be drawn with knitted tubular lattice composites, where energy absorption improves with a higher number of cells to be deformed [35]. 

The role of the connection between the layers is to be further studied. The contact points between the pleated layers of the laminate are essential for the material to perform as a constrained unit under load and prevent immediate sliding of the layers. However, the displacement of the layers during compression shows a relative sliding of the layers. This sliding is within the elastic range of the material and its contact points, since the meso-scale architecture of the material is not destroyed through the extensive loading, and the layers within each sample remain connected. 

Looking at the analysis of entangled materials, the connection between the components’ contact length and permanence of contact seems to be a critical architectural parameter in the material performance [34]. A fundamental difference was found between entangled materials with initial permanent connections between components (epoxy cross-links between fibers) and without rigid connections (loose fibers). The type of stress–strain curves of the two materials is different and indicates an evolution of connection points along loading; the unlinked material starts with low initial stiffness, which increases with deformation as new contact points are created by compression and friction augments. This typically exponential stress–strain curve resembles the curve of L-FMFRP, where stiffness increases with deformation. The material of permanent links goes through a three-step process, starting with elastic behavior, going through a plateau where permanent connections are destroyed and new contact points are constructed, and ending with pure densification of the contact points [34]. This type of curve resembles the curve of the first loading cycle (Figure 5). The evolution of the stress–strain curve in cyclic loading, from linear-like curve to exponentially growing curve, indicates the evolution of the contact points and friction level of the material during increased compression. Contact points between the pleated layers of L-FMFRP are permanent and persist during compression, indicating that these contacts perform within the elastic range of the resin incorporating them. However, we have no indication of the number of contacts and the extension of the contact areas before and after the loading, and it is probable that a certain number of connections are at least partly destroyed during the first cycle of compression. This might explain the resemblance between the curve of the first compression cycles and the typical curves of the permanently linked entangled systems, with a three-step evolution of contact points.

The role of friction in the hysteresis of entangled material was studied previously [36,37], indicating its direct relation to the hysteresis in stretching and bending energy; it was found to be determined by the friction coefficient and average number of contact points between the elements. As L-FMFRP only partially resembles entangled materials, the relevance of its friction model to describe the behavior of our system requires further study. 

## 5. Conclusions

The L-FMFRP material system product resembles a thick panel with highly articulated surfaces of extreme low density. It stands in contrast to typical FRP panels: its section is not uniform and dense, but variable and airy; it has an intricate internal configuration set by the pleats and the partial adherence between the layers. This, in turn, affects and determines the characteristics of the overall resulting element, its stiffness and strength. L-FMFRP blurs the boundaries between material and structure, and thus can be considered an ‘architectured material’, for which the overall macro-scale behavior is related to two underlying scales: the micro-scale, where the density and the direction of the fibers play a principal role in defining the textile’s properties; and the meso-scale, where a complex geometry and a set of internal bonds give rise to a particular, airy, structure. 

The finite deformation properties of L-FMFRP and the hysteresis phenomena demonstrated in the experiments indicate a potential capacity of the material system for energy absorbance. The experiments show that this property is inherent to the material’s architecture, and is affected by parameters such as pleat type and number. Such a capacity for energy absorbance, if further investigated and developed, could prove to be beneficial for architectural cladding applications, as retrofit or for new structures. Other energy absorbent composite structures designed for crashworthiness applications could serve as a reference [26]. While these are mainly implemented in moving structures (transportation), here, another application in architectural structures is suggested. Possible implementation could be a development of L-FMFRP, either as part of a composite sandwich construction or as a single “thick skin” with cellular characteristics. 

An initial estimation is to be carried out to assess the capacities of the L-FMFRP system to withstand the range of loads typically considered for windborne debris, blast mitigation by sacrificial façade systems and general energy dissipation for fluctuating air pressure. Referring to its crashworthiness properties, its characterization should refer to indicators of performance such as the specific energy absorption (SEA), energy absorption (EA) capacity, crush force efficiency (CFE), mean crushing force (MCF) and sound transmission loss (STL) [26]. With that, a better understanding is required as to the capacity of the material system to be enhanced by variation of the fabrication parameters or the association of additional elements to the system. 

The further investigation of L-FMFRP calls for a deeper understanding of the internal physical phenomena of energy dissipation in the system and its determining parameters. Wishing to augment its capacities for energy absorbance, the relation between friction, structure and geometrical pattern is to be evidenced. Monitoring the heat loss and the sound emitted throughout the full loading cycle could indicate the fractional energy loss that takes place. The role of contact points, their evolution along compression cycles, their effect on friction, their relation to the folding pattern and the effect of those on the hysteresis level should be further investigated in order to optimize the energy absorbance of the system. Through further physical experimentations and mathematical modelling, control of the system’s performance could be obtained through the variation of the geometrical folding pattern and raw material properties. 

## Figures and Tables

**Table 1:** Pleating types–second experimental campaign: samples’ data.

Sample Num.	Pleat Type	Num. Pleats Layer 1	Num. Pleats Layer 2	Final Dimensions (mm)
1	simple	3	3	180 L/195 W/35 H
6	Simple	1	1	205 L/200 W/30 H
5	Full	1	1	330 L/195 W/40 H
4	Full	2	1	300 L/190 W/45 H

## Data Availability

The data that support the findings of this study are openly available in the Figshare repository: https://doi.org/10.6084/m9.figshare.18515129 (accessed on 25 January 2022).
